# Role of HLA in Hematopoietic Stem Cell Transplantation

**DOI:** 10.1155/2012/680841

**Published:** 2012-10-02

**Authors:** Meerim Park, Jong Jin Seo

**Affiliations:** ^1^Department of Pediatrics, College of Medicine, Chungbuk National University, Cheongju, Republic of Korea; ^2^Department of Pediatrics, Asan Medical Center Children's Hospital, University of Ulsan College of Medicine, Seoul, Republic of Korea

## Abstract

The selection of hematopoietic stem cell transplantation (HSCT) donors includes a rigorous assessment of the availability and human leukocyte antigen (HLA) match status of donors. HLA plays a critical role in HSCT, but its involvement in HSCT is constantly in flux because of changing technologies and variations in clinical transplantation results. The increased availability of HSCT through the use of HLA-mismatched related and unrelated donors is feasible with a more complete understanding of permissible HLA mismatches and the role of killer-cell immunoglobulin-like receptor (KIR) genes in HSCT. The influence of nongenetic factors on the tolerability of HLA mismatching has recently become evident, demonstrating a need for the integration of both genetic and nongenetic variables in donor selection.

## 1. Introduction

Allogeneic hematopoietic stem cell transplantation (HSCT) has been established as a mode of curative therapy for hematologic malignancies and other hematologic or immune disorders. Hematopoietic stem cell donor selection has been almost exclusively based on selecting an human leukocyte antigen (HLA) identical donor or near-identical donor; however, not all patients are able to find a suitable donor. Advances in HLA testing and matching and understanding donor selection factors are therefore important to improve outcomes of unrelated donor (UD) HSCT. HLAs can elicit an immune response either by presentation of variable peptides or by recognition of polymorphic fragments of foreign HLA molecules. HLA disparity has been associated with graft failure, delayed immune reconstitution, graft-versus-host disease (GVHD), and mortality. Since many patients lack HLA-matched donors, current research is focused on the identifying permissible HLA mismatches. Recently, extensive research has accumulated evidence on the role of each HLA locus mismatch on clinical outcome for UD HSCT, making it easy to search for and select a partially matched donor [1, 2].

In this paper, we will focus on the current understanding of HLA typing and its clinical implications on UD HSCT.

## 2. HLA Typing

HLA class I and II loci are the most polymorphic genes in the human genome, with a highly clustered and patchwork pattern of sequence motifs [3]. Each individual carries 10 to 12 genes that encode the HLA-A, -B, -C, -DR, -DQ, and -DP. Most of these genes are highly polymorphic, ranging from 13 (HLA-DRB4) to 699 (HLA-B) alleles per locus [4]. Extensive allelic diversity has made, and continues to make, high-resolution HLA-DNA typing very challenging. Over the past three decades, the remarkable extent of allelic diversity at these loci has been shown by molecular genetic analyses, made possible by the development of recombinant DNA technology, chain-termination Sanger sequencing, and PCR amplification [3].

Initially, HLA-DNA typing involved restriction fragment length polymorphism (RFLP) analysis, but this approach had many limitations in terms of workflow and resolution and represented at best a complement to, rather than a replacement for, serological typing [5]. The development of PCR in 1985 allowed for the amplification of the polymorphic exons of the HLA class I and II genes and for the analysis of polymorphic sequence motifs with sequence-specific oligonucleotide (SSO) hybridization probes. Currently available methods to identify specific polymorphisms or nucleotide motifs include SSO probe hybridization, sequence-specific primer (SSP) amplification, sequencing-based typing (SBT), and reference-strand-based conformation analysis [3, 6]. Both PCR-SSP and PCR-SSO rely on the use of oligonucleotide primers or probes to react and/or detect specific and previously known polymorphic sequence motifs present within the amplified HLA-allele fragment. A major disadvantage is that such methods rely on the screening of a limited number of previously known polymorphisms. Therefore, when a novel allele is present a sample, mistyping can occur, depending on whether the allele possesses a different polymorphism or different arrangement of known polymorphisms. However, SBT uses generic oligonucleotide primers directed towards conserved regions of a locus to amplify the polymorphic exons of all alleles. Although SBT is able to detect previously unknown HLA alleles, it is not entirely capable of resolving novel arrangements of known polymorphisms, a limitation known as ambiguity. This problem can be overcome by separating the alleles by groups or allele-specific PCR, cloning, or by the use of conformational techniques. Conformational methods, such as the Reference-Strand-mediated Conformational Analysis (RSCA), have shown to achieve high-resolution results without the ambiguities seen in the previously mentioned methods [7].

HLA-typing methods convey certain advantages and present various limitations. Matching by high-resolution HLA typing, a more recent and sophisticated method, certainly reduces the risk of immune complications, namely, graft rejection and GVHD along with increased chance of finding a suitable donor [2]. As such, the choice of method is dependent on the intended application and on establishing an appropriate balance of what level of resolution is needed with regards to speed of typing, cost, and human intervention [8].

## 3. Effect of HLA on Clinical Outcomes after HSCT

### 3.1. Number of HLA Mismatches

Advances in HLA-typing techniques allowing better matching of donor-to-recipient have improved the prognosis of HSCT. A recent prospective study investigating outcomes after transplant with 10/10 allelic-matched unrelated donors (MUDs) and HLA-identical sibling grafts for patients with standard-risk hematological malignancies showed that overall survival, disease-free survival, transplantation-related mortality (TRM), relapse, and acute GVHD were not dependent on donor type [9]. The similar outcome values for different donor types suggest that well-selected UDs can perform as well as HLA-identical sibling donors. Immune genetic disparity in the donor-recipient pair is associated with a worse patient outcome, mainly due to the high incidence of transplantation-related complications. A direct assessment of the number of HLA mismatches between the donor and the recipient has highlighted its great importance in UD HSCT. As the number of class I and II HLA mismatches increases, the risks of graft failure, GVHD, and mortality increase [10–12]. Indeed, a recent analysis by the Center for International Blood and Marrow Transplant Research (CIBMTR) on patients with hematological malignancies, mainly transplanted with bone marrow cells, has shown that, as compared to patients transplanted from a donor matched at the allelic level for HLA-A, -B, -C, and -DRB1, patients given an allograft from a donor with a single antigenic or allelic disparity had an increased risk of both acute GVHD and TRM [2]. Disparities at two or more loci compounded this risk. 

### 3.2. Permissible Mismatches

The need to broaden the availability of UD HSCT for patients who lack a matched donor has provided a rationale to define permissible HLA mismatches. The most important HLA loci influencing posttransplant outcome of patients given HSCT from UDs are HLA-A, -B, -C and -DRB1 [13, 14]. There have been several large-scale analyses on the role of each HLA locus in non-T-cell-depleted UD HSCT (Table 1). The Japan Marrow Donor Program (JMDP) showed the effect of matching HLA class I alleles on the development of severe acute GVHD and the importance of HLA-A and -B allele matching for better survival [10, 15]. The Fred Hutchinson Cancer Research Center (FHCRC) and the US National Marrow Donor Program (NMDP) reported the importance of HLA class II matching to prevent GVHD and to increase survival [13, 16]. An analysis of NMDP in 2004 indicated that HLA-A allele level mismatching, HLA-B serological mismatching, and DRB1 mismatching are significant risk factors for severe acute GVHD and that disparity in HLA class I and/or HLA-DRB1 increases the incidence of mortality [14]. An analysis of NMDP published in 2007 showed that the impact of HLA-A or -DRB1 mismatch on overall survival was more marked than a mismatch at HLA-B or -C [2]. And recent analysis of Korean data showed the importance of HLA-B and -C locus matching for better survival [11]. However, the above-mentioned reports, as well as others, have produced considerable conflicting results on the causal role of HLA mismatch locus on clinical outcomes. 

The significance of HLA mismatching may be related to population-based locus- and allele-specific differences that distinguish ethnically diverse transplant donors and recipients. The International Histocompatibility Working Group (IHWG) studied the impact of individual locus mismatches in different populations [17]. The authors found that a single HLA-A mismatch was poorly tolerated in JMDP transplant recipients, but less detrimental in the non-JMDP population. Conversely, mismatches at HLA-C were well tolerated among the JMDP patients, but poorly tolerated among non-JMDP patients. One explanation for this may be differences in the actual allele mismatches in these separate populations. Morishima et al. [18] reported that the most frequent mismatch found in Japanese patients was HLA-A*0201 and HLA-A*0206 and that this mismatch was deleterious. By contrast, the most common HLA-A*02 mismatch in Caucasians was found to be HLA-A*0201 and HLA-A*0205, and an adverse relationship between this mismatch and transplantation outcomes was not found. The identification of a nonpermissive HLA-allele mismatch combination indicates that the ethnic diversity of the recipient and donor can translate into molecular differences based on HLA alleles, indicating that it is essential to reconcile differences in HLA risk observed among ethnically diverse transplant groups. Analysis of HLA-DPB1 mismatches in this way has lead to interesting findings [19, 20]. Crocchiolo et al. [21] reported a significantly higher 2-year survival in transplants with permissive as compared to nonpermissive HLA-DPB1 mismatches (54.8% versus 39.1%, *P* = 0.005). Similarly, Zino et al. [20] found a significantly higher risk of mortality in patients with nonpermissive DPB1 mismatches compared to those without such mismatches.

### 3.3. HLA-DQ and HLA-DP

The importance of HLA-A, -B, -C, and -DR in HSCT has been well described, whereas there have been conflicting results as to the clinical significance of HLA-DP and -DQ. Less than 20% of transplants matched for HLA-A, -B, -C, -DRB1, and -DQB1 are also compatible for HLA-DPB1, due to the very weak linkage disequilibrium existing between the DR/DQ loci and the DP locus. Therefore, over 80% of unrelated transplants are performed across the HLA-DPB1 barrier [2, 22]. Furthermore, the low frequency of fortuitous HLA-DP matching hinders a precise analysis of the true independent effects of HLA-DP mismatching except in cases of very large numbers of transplants. Early investigations were conflicting as to the significance of HLA-DP as a classical transplantation determinant. In a recent analysis of 627 HLA-identical sibling transplants, of which 30 were HLA-DP-mismatched due to recombination, HLA-DP mismatch was an independent risk factor for GVHD [23]. Furthermore, Schaffer et al. [24] reported that mismatching for HLA-DP was a risk factor for increased mortality compared to DP matching. Most studies now agree that HLA-DQB1 does not need to be considered in a well-matched donor [10], but evidence supports that there may be an additive effect of a DQB1 mismatch if a mismatch at another locus is present [25]. Taken together, roles of the HLA-DQ and DP loci remain not fully elucidated. However, previous results suggest that when patients have a choice of equivalently matched donors, selection of an HLA-DQB1-matched donor over a mismatched donor may decrease posttransplant complications.

### 3.4. Level of HLA Disparity

The level of HLA disparity (antigenic or allele level) affects HSCT outcome differently [26–28]. Sequence analyses show that antigenic disparity is frequently associated with more than ten amino acid substitutions in HLA molecules, which can be easily recognized by immunocompetent cells, thereby stimulating an immune response [26]. Allele level disparity most frequently concerns only one or a few amino acid substitutions, which should produce weaker immune stimulations. A linear increase in the number of amino acid substitutions in the disparate HLA molecule may cause significant deleterious effects or be irrelevant in HSCT [26, 29]. However, there are conflicting data concerning the value of selecting an allelic mismatch over an antigenic mismatch. According to Lee et al. [2], there were no significant differences in survival depending on whether the mismatch was allelic or antigenic, except at HLA-C, in which an antigenic mismatch increased transplant risks while an allelic mismatch did not. Similarly, a single-center study from Seattle could not find any apparent difference between allele and antigen mismatches with respect to the number of deaths from transplants, suggesting that donors with a single HLA allele of antigen mismatch may be used for HSCT when a fully MUD is not available for patients with severe diseases not permitting time for a lengthy search [25]. However, the NMDP study found that antigenic mismatch was associated with higher mortality compared to allelic mismatch [14]. They indicated that selection of donors with high-resolution mismatches over those with low-resolution mismatches may lower the rate of posttransplant complications. The analysis of large transplant populations with a diversity of mismatches is needed to further define potential differences between allele and antigen mismatches in post-HSCT complications.

### 3.5. Tolerable Mismatches

Although HLA-identical donors are now known to be the gold standard, using a donor with a single-allele mismatch has been associated with an equally favorable outcome in certain situations. According to the report of Teshima et al. [30], reduced intensity conditioning (RIC) transplantation from a two- to three-loci-mismatched donor resulted in poor outcome, as shown in conventional HSCT. However, the 2-year overall survival after one-locus-mismatched RIC transplantation was comparable with that of HLA-matched RIC transplantation in high-risk malignancies. In a study of T-cell-depleted RIC transplants, there was no significant difference in overall survival between matched or one-antigen-mismatched grafts [31]. A recent report from the United Kingdom, in recipients of T-cell-depleted RIC transplantation protocols using Alemtuzumab, showed that transplant outcomes were similar between HLA-matched and, mismatched pairs [32]. As listed above, in settings of T-cell depletion and/or RIC transplantation, the impact of HLA matching may differ and these conditions require further investigation.

### 3.6. Importance of Disease Stage

It is important to note that the effect of a single-allele mismatch may vary with the underlying diagnosis. In a recent publication on 948 donor-recipient pairs at the FHCRC, it was found that a single-allele mismatch conferred a higher risk of death, but only for low-risk patients, defined as those with chronic myeloid leukemia (CML) within 2 years of diagnosis [25]. By contrast, a single-allele mismatch had no effect on survival among higher-risk patients, such as those with more advanced CML, acute leukemia, or myelodysplastic syndrome. Similar outcomes are reported in a recent report from an Italian group [33]. When only a single HLA mismatch (9/10 matched pairs) was present, the mortality risk was higher than among 10/10 matched pairs in patients transplanted with acute leukemia in the first CR (early stage disease), but not in patients with advanced diseases. These results suggest that the potential benefit of HLA matching was offset by the negative impact of advanced disease. Therefore, if a donor search is highly unlikely to yield matched donors in the early phases of disease, the increased mortality associated with a longer time interval from diagnosis to transplantation must be weighed carefully against the increased mortality associated with earlier HSCT with a mismatched donor, as well as against the chance of disease progression during the prolonged donor search.

## 4. The Role of Anti-HLA Antibodies

Donor-specific anti-HLA antibodies (DSHAs) have been implicated in graft rejection in solid-organ transplantation, but their role in allogeneic HSCT remains under investigation [34–36]. Controversy exists as to whether DSHAs actually mediate graft rejection or if they are surrogate markers for cellular immunity that cause graft failure [37]. DSHAs cause graft failures in animal models of allogenic HSCT, mainly because the cognate HLA antigens are expressed on hematopoietic stem cells and hematopoietic precursors [38].

The complement-dependent microlymphocytotoxicity assay (CDC) has been the standard method for the detection of anti-HLA antibodies for the last 30 years [39]. The recent introduction of solid-phase assays enabled a reassessment of the role of both HLA class I and II antibodies in organ rejection. Spellman et al. [40] tested archived pretransplantation sera from graft failure patients and a matched-control cohort to evaluate the role of DSHAs in UD HSCT. The presence of DSHAs was significantly associated with graft failure (odds ratio = 22.84; 95% CI, 3.57–infinity), indicating that the presence of pretransplantation DSHAs in recipients of UD HSCT should be considered in donor selection. Similarly, Ciurea et al. [41] found that DSHAs were associated with a high rate of graft rejection in patients undergoing haploidentical HSCT. On the basis of the previously mentioned findings, DSHA identification should be performed in HSCT settings where HLA matching is not complete [42]. Immunoadsorption and plasmapheresis could be considered to desensitize the recipient when no alternative donor is available.

## 5. Killer Immunoglobulin-Like Receptor (KIR) Ligand Incompatibility

Natural killer (NK) cells and subpopulations of T cells express NK cell receptors. The activity of NK cells is controlled by the recognition of HLA class I molecules on the target cells by NK cell inhibitory and activating receptors [43, 44]. Depending on the type of KIR, ligation by HLA can stimulate or inhibit the ability of NK cells to kill foreign cells, including tumor cells [45]. The coexistence of the incompatibility of both types on the same HLA molecules makes it difficult to show the advantages of KIR-ligand mismatches clearly. The strong immune reactions provoked by T-cell recognition elements on incompatible HLA molecule can probably override the favorable effect of the simultaneous KIR-ligand mismatch [46]. In fact, Farag et al. [47] investigated the effect of KIR-ligand mismatching on the outcome of UD HSCT in the T-replete setting. In that study, patients who received grafts from donors mismatched at the KIR ligand and at HLA-B and/or C but matched at the KIR ligand had similar rates of TRM, treatment failure, and overall mortality. By contrast, Giebel et al. [48] investigated UD HSCT in 130 patients with acute myeloid leukemia (AML), acute lymphoblastic leukemia (ALL), or CML, who received unmanipulated grafts. The results of that study showed that transplant from KIR-incompatible donors resulted in enhanced overall survival, decreased disease relapse, and increased probability of disease-free survival. When myeloid leukemia patients were selected for analysis, these effects became more prominent, suggesting that patients with myeloid malignancies were more responsive to treatment. More recently, Cooley et al. [49, 50] analyzed the outcomes of 1,409 patients, taking into account the role of KIR-gene variability. Donor KIR genotype influenced transplantation outcomes for patients with AML but not for those with ALL. Compared to donors without KIR mismatches, donors having KIR mismatches showed reduced incidences of relapse and improved disease-free survival. Furthermore, KIR-ligand incompatibility in the graft-versus-host direction in haplotype-mismatched transplants suggests a possible clinical benefit as it may allow early recovery of donor alloreactive NK cells with enhanced antileukemia activity in AML [51].

If KIR mismatch results in graft versus tumor (GVT) effects, one may assume that several mismatches would result in further enhances in the GVT effect. Previous transplant studies commented upon the impact of numerous mismatches compared to one mismatch. Clausen et al. [52] demonstrated that relapse risk was decreased in patients who underwent HLA-identical sibling HSCT who both received high NK cell dose and lacked at least one HLA-B or HLA-C ligand to a present donor's inhibitory KIR. In that study, transplants with more than two different activating donor KIRs were associated with an increased risk for nonrelapse morality. Similarly, Willemze et al. [53] reported that a higher number of HLA disparities resulted in a decreased incidence of relapse in patients who received umbilical cord blood transplantation.

Collectively, it is clear that the exploitation of NK cell alloreactivity as a therapeutic advantage in HSCT is promising, and certain patients with myeloid malignancies have benefited from allogeneic HSCT. KIR genotyping of several best HLA-matched potential UDs may change clinical practice in the future [54].

## 6. Conclusion

A donor's HLA match status should be considered to help the physician and patient in transplantation-related risk assessment and in planning treatment options based on those risks. The benefits of high-resolution HLA class I and II typing have been well demonstrated, particularly for posttransplant survival. The current gold standard is a donor matched for 8/8 alleles; however, it is clear that mismatches may be tolerated with regards to survival in some transplant settings and that evidence for permissive mismatches exists. Permissiveness depends not only on the potential adverse effects of HLA mismatches, but also on the urgency of the HSCT, the desirable GVT effect, and the potential efficacy of the alternative therapy available for the patient. Further knowledge on DSHAs, NK cell alloreactivity, and KIR receptors will aid HSCT in becoming safer and more efficacious.

## Figures and Tables

**Table 1:** Effect of HLA mismatching on survival.

Study	Mismatched HLA locus			
	A	B	C	DRB1
Petersdorf et al. [13]	Merged A, B, and C Decreased			Decreased
Morishima et al. [10]	Decreased	Decreased	None	None
Flomenberg et al. [14]	Decreased	Decreased	Decreased	Decreased
Lee et al. [2]	Decreased	None	Decreased	Decreased
Park et al. [11]	None	Decreased	Decreased	None
